# Audiometric evaluation in different clinical presentations of otitis media^☆^

**DOI:** 10.1016/j.bjorl.2023.101359

**Published:** 2023-11-10

**Authors:** Ana Luiza Papi Kasemodel de Araújo, Francisco Polanski Cordeiro, Rafael da Costa Monsanto, Norma de Oliveira Penido

**Affiliations:** a Universidade Federal de São Paulo – Escola Paulista de Medicina (UNIFESP), São Paulo, SP, Brazil; b Otopathology Laboratory – University of Minnesota, Minneapolis, MN, USA

**Keywords:** Otitis media, Hearing loss, Tinnitus, audiometry

## Abstract

**Objectives:**

To assess the hearing thresholds in acute otitis media, otitis media with effusion and chronic otitis media (non-suppurative, non-cholesteatomatous suppurative and cholesteatomatous) and to compare the hearing outcomes with non-diseased ears (in bilateral cases) or contralateral healthy ears (in unilateral cases), since hearing loss is the most frequent sequel of otitis media and there is no previous study comparing the audiometric thresholds among the different forms of otitis media.

**Methods:**

Cross sectional, controlled study. We performed conventional audiometry (500–8000 Hz) and tympanometry in patients with otitis media and healthy individuals (control group). Hearing loss was considered when the hearing thresholds were > 25 dBHL.

**Results:**

Of the 112 patients diagnosed with otitis media (151 ears), 48 were men (42.86%) and 64 were women (57.14%). The average age was 42.72 years. Of those, 25 (22.32%) were diagnosed as AOM, 15 (13.39%) were diagnosed with OME and the remaining 72 (63.28%) were diagnosed with COM (non-suppurative COM, n = 31; suppurative COM, n = 18; cholesteatomatous COM, n = 23). As compared with controls, all forms of otitis media had significantly higher bone-conduction thresholds (500–4000 Hz). Conductive hearing loss was the most frequent type of hearing loss (58.94%). However, the number of patients with mixed hearing loss was also relevant (39.07%). We noted that the presence of sensorioneural component occurred more frequently in 1) Higher frequencies; and 2) In groups of otitis media that were more active or severe in the inflammatory/infective standpoint (AOM, suppurative COM and cholesteatomatous COM).

**Conclusion:**

All types of otitis media, even those with infrequent episodes of inflammation and otorrhea, had worse bone conduction thresholds as compared with nondiseased ears (*p* < 0.01). We observed worse hearing outcomes in ears with recurrent episodes of otorrhea and in ears with AOM, especially in high frequencies.

## Highlights

• Otitis a common disease with several complications, specially hearing loss.
• There is no previous study comparing hearing in different presentations of otitis.
• We observed sequelae in the various groups, even in acute cases after resolution.

## Introduction

Otitis media is defined as the presence of an inflammatory process in middle ear topography and comprises a spectrum of related diseases covering lesions in several phases: acute, subacute, and chronic.^1^ According to Paparella et al.,^1^, ^2^, ^3^ otitis media occurs as a continuum of associated diseases, in which a chronic middle ear inflammatory process can lead to tissue changes and structural lesions progressively.

It is estimated that its incidence is 10.85% or 709 million cases per year worldwide for Acute Otitis Media (AOM) and 31 million new cases per year for Chronic Otitis Media (COM), and 22.6% of these cases occur in children under 5 years of age.^4^, ^5^ The overall incidence of suppurative COM is estimated at 4.8 new cases per 1000 people/year, with a prevalence of around 65–300 million individuals.^4^, ^6^

Due to the high incidence and prevalence rates, the non-lethal complications of otitis media (hearing loss, tinnitus, vestibular symptoms) should receive special attention, as they cause great morbidity to patients. About 60% of people with Suppurative COM attend hearing loss worldwide, which corresponds to 39–200 million individuals.^5^

In the various presentations of otitis media, the most common type of hearing loss is conductive, either due to the presence of effusion, tympanic membrane perforation or ossicular changes. However, in recent decades, sensorineural hearing loss secondary to the inflammatory processes of the middle ear has been demonstrated.^7^, ^8^, ^9^ Inflammatory products and toxins from the middle ear cross the membrane of the round window, mainly affecting the basal turn of the cochlea,^10^ which can lead to inflammatory injury of its sensory and neural elements. The most severe location of these cochlear changes (basal turn) corresponds tonotopically to the most affected frequencies (acute frequencies).^7^, ^10^, ^11^

In general, all otitis media present with some degree of hearing loss, temporary or permanent. Despite such a common complaint, there is no study comparing the auditory thresholds in the various clinical presentations of otitis media, which is the main objective of our research.

## Methods

The study was of the individual cross sectioned study (level 2). We selected patients from the otology outpatient clinic and the otorhinolaryngology emergency department of our institution. We divided the patients into non-suppurative COM, suppurative COM, cholesteatomatous COM (suppurative and non-suppurative forms), Otitis Media with Effusion (OME) and Acute Otitis Media (AOM). Ears with otitis media were compared with ears of healthy individuals from the otological point of view. All patients and individuals in the control group were selected after applying the inclusion and exclusion criteria.

We included patients with 1) Diagnosis of otitis media, made on an outpatient basis or in the emergency room by an ENT physician; 2) Patients over 10 years of age; 3) Patients with AOM of up to 5 days of evolution.

We excluded patients who had: 1) History of otologic surgery or placement of ventilation tube; 2) Cognitive alterations; 3) Syndromic diseases or with external, middle or internal ear malformations; 4) Chronic exposure to noise; 5) History of primary cancers or metastases to the temporal bone; 6) History of systemic chemotherapy or radiotherapy in the head and neck region; 7) Autoimmune diseases; 8) History of exposure to aminoglycosides or other potentially ototoxic medications; 9) Cases of diabetes mellitus or other systemic diseases that may compromise the auditory thresholds; 10) History of head trauma or traumatic perforations of the Tympanic Membrane (TM).

We selected the individuals from the control group using a non-probabilistic sampling method (“convenience sample”). We applied the same exclusion criteria for this group and included only individuals with air and bone <25 dBNA and absence of bone air GAP. Only individuals and patients who signed and agreed with the informed consent form participated in the study.

Computed tomography of temporal bones was performed in patients with chronic otitis media to classify the type of clinical presentation. Some cases of OME and non-suppurative COM were submitted to computed tomography for better diagnostic elucidation.

We performed conventional tonal audiometry (500–8000 Hz), vocal audiometry with discrimination scores and speech recognition thresholds (SRTs) evaluation and imitanciometry (when the tympanic membrane was intact) in patients with otitis media and in the control group. We considered hearing loss when the auditory thresholds were > 25 dBNA. Audiometric examinations were performed by an experienced audiologist.

Statistical analysis was performed with R version 3.5.1. for Windows. To compare the groups of otitis media with each other, as well as to compare the various groups of otitis media with the control group, the Kruskal-Wallis test and the Dunn-Bonferroni post hoc test were used. We used the same statistical tests to analyze the statistical significance of the presence of tinnitus between the groups. Spearman's correlation was used to correlate numerical variables. Values of *p* < 0.05 determined the statistical significance.

The study was approved by the Research Ethics Committee (number 0364/2017) and consent was obtained from all the participants that agreed to join in the research.

## Results

### Demographics

After application of our inclusion and exclusion criteria, our otitis media group comprised of 116 patients. We analyzed each ear individually, which resulted in a total of 151 ears studied in the otitis media group – of the 116 cases, 35 (30.17%) were bilateral otitis and 81 cases (69.83%) were unilateral otitis. These ears were compared with 122 ears of 63 otologically normal and healthy individuals, with no history of otitis, hearing loss or otological/brain trauma.

**Figure 1:**
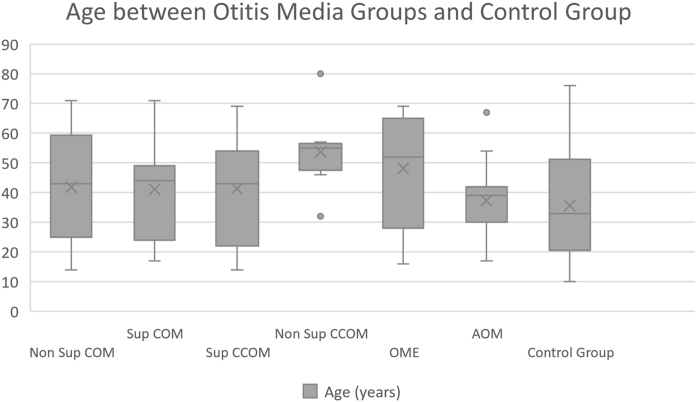
Age comparison between otitis media groups and control group.

Of the 116 patients with otitis media, 67 (57.76%) were female and 49 (42.24%) were male. The control group consisted of 23 men (36.5%) and 40 women (63.5%). The ages of each group are described in Table 1 and Fig. 1.

**Table 1:** Mean age of the patients in our study and control groups.

Age	Groups						
	Non suppurative COM	Suppurative COM	Suppurative CCOM	Non suppurative CCOM	OME	AOM	Control group
Average	43.12	42.47	41.32	53.67	47.71	37.75	35.6
Median	44	39	43	55	51.5	37.5	33
Standard-deviation	18.03	18.65	17.91	12.6	18.02	10.95	16.98
Max‒min values	14‒71	14‒71	14‒69	32‒80	16‒69	17‒67	10‒76

Regarding the distribution of patients among the groups of otitis media, 31 (26.72%) had non-suppurative COM; 19 patients (16.38%) had non-cholesteatomatous suppurative COM; 24 patients (20.69%), cholesteatomatous COM; 14 patients (12.07%), OME and 28 patients (24.14%), AOM. Regarding the number of ears, 38 (25.17%) were classified as non-suppurative COM; 27 (17.88%), as suppurative COM; 28 (18.54%), as cholesteatomatous COM (of these, 19 were of the suppurative subtype and 9 were of the non-suppurative subtype); 27 (17.88%) were classified as OME and 31 (20.53%) were classified as AOM.

**Figure 2:**
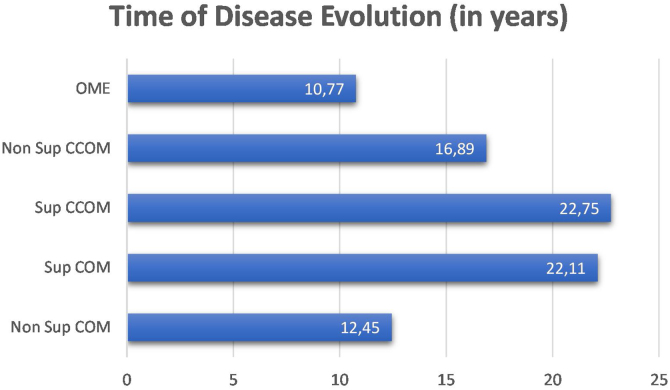
Time of disease evolution among the chronic cases of otitis media.

We did not observe a correlation between disease duration and bone thresholds of chronic cases of otitis media (OMCs and OMEs) (*p* = 0.1362). We compared the disease duration in chronic cases of otitis media and did not observe a statistically significant difference between them (*p* = 0.191). Nevertheless, it is observed in the graph in Fig. 2 that the suppurative form of the cholesteatomatous COM and the suppurative COM have a longer time of disease evolution than the other otitis media.

### Clinical evaluation

**Figure 3:**
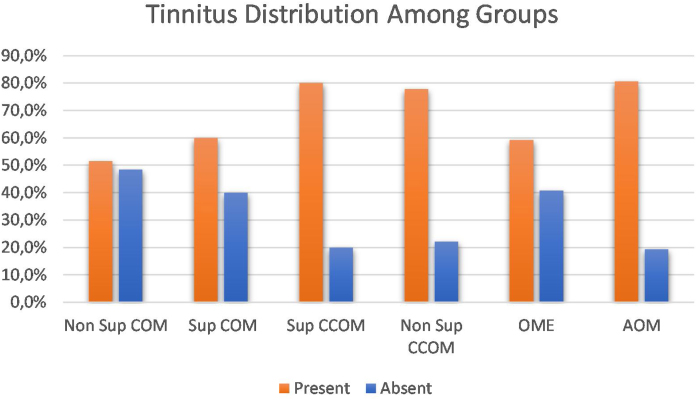
Tinnitus distribution in percentages among otitis media groups.

Regarding the symptoms reported by the patients, hypoacusis was the main complaint – reported by 93.1% of the patients analyzed. Tinnitus was the second most prevalent complaint, present in approximately 69% of cases. We compared the prevalence of tinnitus among the different groups of otitis media and observed a significant difference between them (*p* = 0.043). When comparing the groups with each other, we observed a higher prevalence of tinnitus in the COM groups suppurative x non-suppurative COM (*p* = 0.01); Non-suppurative COM (*p* = 0.03) and Non-suppurative COM × AOM (*p* = 0.01). There was no correlation between disease time and tinnitus (*p* = 0.717) (Fig. 3).

### Audiometric evaluation

Audiometric evaluation revealed that most patients (58.28%) had conductive hearing loss. About 40.4% of the patients had mixed hearing loss and 1.32% had pure sensorineural hearing loss. Table 2 discriminates the types of hearing loss in each group.

**Table 2:** Types of hearing loss.

Groups	Types of hearing loss in each group of otitis media			
	Conductive	Mixed	SNHL	Total ears
Non Sup COM	28 (73.68%)	10 (26.31%)	0 (0.0%)	38 (100%)
Sup COM	12 (44.4%)	15 (55.6%)	0 (0.0%)	27 (100%)
Sup CCOM	10 (52.53%)	9 (47.37%)	0 (0.0%)	19 (100%)
Non Sup CCOM	5 (55.5%)	4 (44.4%)	0 (0.0%)	9 (100%)
OME	19 (70.4%)	8 (29.6%)	0 (0.0%)	27 (100%)
AOM	14 (45.16%)	15 (48.4%)	2 (6.45%)	31 (100%)
Total	88 (58.28%)	61 (40.4%)	2 (1.32%)	151 (100%)

**Figure 4:**
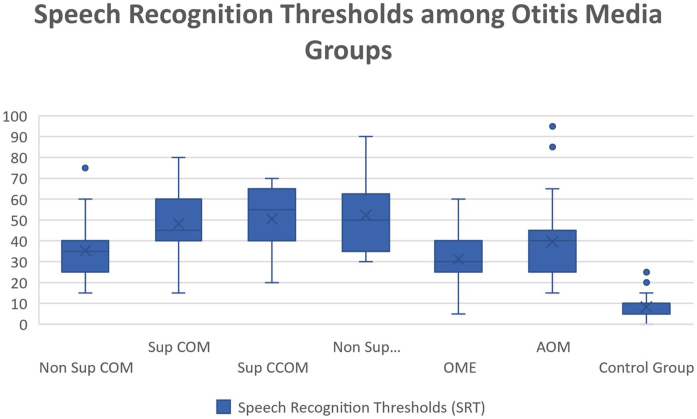
Boxplot illustrating otitis media’s and control group’s SRT values.

Regarding vocal audiometry, we could observe a statistically significant difference in SRT when comparing the ears of the otitis media groups with the ears of the control group (*p* < 0.001). When comparing the groups of otitis media, there was statistical significance between the suppurative COM × OME (*p* = 0.01); suppurative COM × non-suppurative COM (*p* = 0.04) and suppurative COM × OME (*p* = 0.01). Although we did not find significant difference when comparing all groups of otitis media, we observed that the worst thresholds of SRT were suppurative cholesteatomatous COM, followed by non-suppurative cholesteatomatous COM, followed by suppurative COM, followed by non-suppurative COM. Thus, SRT was decreasing, in this order: Suppurative CCOM > non-suppurative CCOM cholesteatomatous COM > non-suppurative colesteatomatous COM > suppurative COM > AOM > non-suppurative COM > OME (Table 3 and Fig. 4).

**Table 3:** Speech recognition thresholds values (in dBHL) between otitis media and control groups.

SRT							
	Non Sup COM	Sup COM	Sup CCOM	Non Sup CCOM	OME	AOM	Control Group
Average	35,4	48,15	50,53	52,22	31,3	39,52	8,36
Median	35	45	55	50	30	40	10
Standard-deviation	12,32	15,7	13,95	17,5	12,52	18,2	5,14
Min‒max values	15‒75	15‒80	20‒70	30‒90	5‒60	15‒95	0‒25

**Figure 5:**
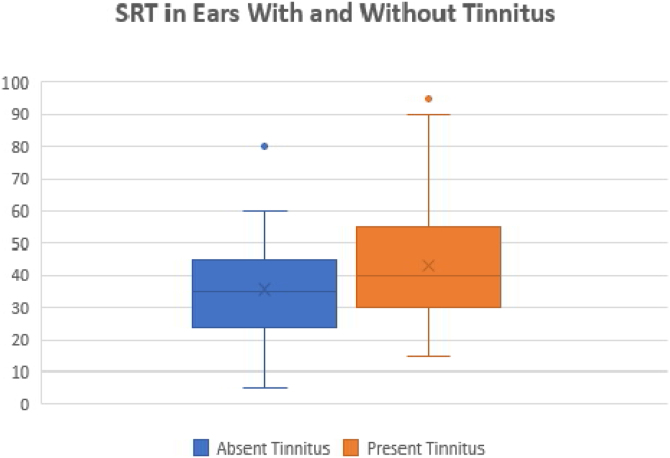
Difference between SRT in ears with and without tinnitus.

We also compared the SRTs between the ears with and without tinnitus. We observed a statistically significant difference between these ears (*p* = 0.01). The ears with tinnitus presented mean and median SRTs values higher than the ears without tinnitus (mean 43.17 dB and median 40 dB versus mean 35.7 and median 35 dB, respectively) (Fig. 5).

Regarding speech discrimination, we observed a significant difference when comparing the discrimination scores between all groups of otitis media and the control group (*p* < 0.001). However, it was not possible to observe statistical significance between the OME group versus the control group (there was statistical significance among the other groups of otitis media versus control group, with *p* < 0.05). When comparing the groups of otitis media with each other, we observed: 1) Worse discrimination scores were found in the suppurative COM group, when comparing it with OME (*p* = 0.002) and AOM (*p* = 0.03) and 2) worse discrimination scores in the non-suppurative COM Group, when comparing it with OME (*p* = 0.03).

**Figure 6:**
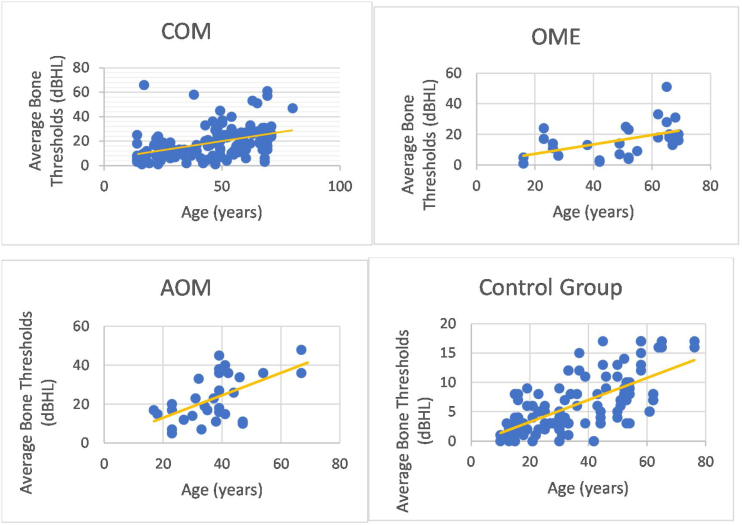
Bone conduction thresholds in the otitis media and control groups according to their age.

We could observe a positive correlation when analyzing the age factor and bone auditory thresholds. In other words, bone thresholds increased with advancing age in patients with COM (non-suppurative, suppurative and cholesteatomatous), in patients with OME, with AOM and in individuals in the control group (however, it is noted that the ears of the control group did not present thresholds above 25 dBNA) (Fig. 6).

**Figure 7:**
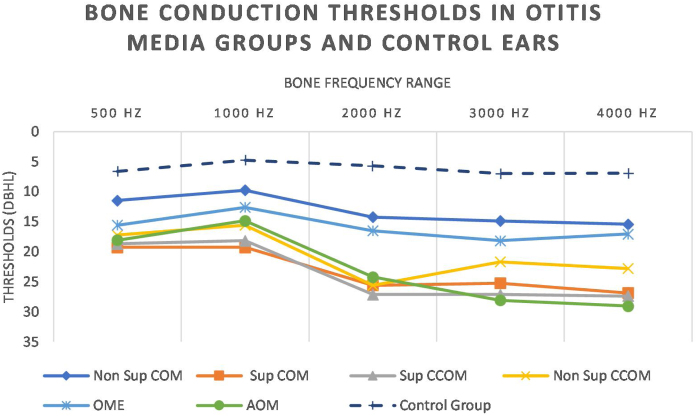
Bone conduction thresholds in otitis media and control groups.

Regarding tonal audiometry, we observed worse bone thresholds in all groups of otitis media when compared with the ears of the control group (*p* < 0.001), in all frequencies (500–4000 Hz) (Fig. 7).

It is noted that the thresholds are worse at the highest frequencies. In addition, it is possible to observe that the most active otitis from the infectious point of view (Cholesteatomatous COM, suppurative COM and AOM) have worse bone thresholds, more pronounced in acute frequencies. Likewise, it is observed that the ears with AOM are with more intense sensorioneural losses than all other groups of otitis media at frequencies of 3000–4000 Hz. Table 4 discriminates the significant differences between bone pathway thresholds in each frequency in the otitis media groups.

**Table 4:** Statistically significant differences in BC thresholds between otitis media groups.

Comparison between ears of otitis media groups	500 Hz	1000 Hz	2000 Hz	3000 Hz	4000 Hz
Sup COM × Non Sup COM	*p* = 0.005	*p* = 0.004	*p* = 0.007		*p* = 0.02
Sup CCOM × Non Sup COM	*p* = 0.017		*p* = 0.02		*p* = 0.041
Sup COM × OME		*p* = 0.03			
AOM × Non Sup COM			*p* = 0.009	*p* < 0.001	*p* < 0.001
AOM × OME				*p* = 0.02	*p* = 0.002

**Figure 8:**
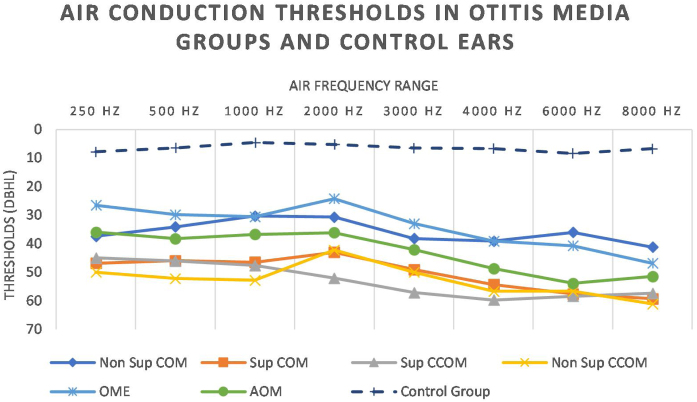
Air conduction thresholds in otitis media and control groups.

We also observed worse air thresholds in all ears with otitis media, when compared with the ears of the control group (*p* < 0.001), in all frequencies (250–8000 Hz) (Fig. 8 and Table 5).

**Table 5:** Statistically significant differences in air conduction thresholds between otitis media groups.

Comparison between ears of otitis media groups	250 Hz	500 Hz	1000 Hz	2000 Hz	3000 Hz	4000 Hz	6000 Hz	8000 Hz
Sup COM × Non Sup COM			*p* = 0.049				*p* = 0.01	
Sup CCOM × Non Sup COM			*p* = 0.04	*p* = 0.02				
Non Sup COM × OME	*p* = 0.03							
Sup COM × OME	*p* = 0.002	*p* = 0.003		*p* = 0.006				
Sup CCOM × OME	*p* = 0.005	*p* = 0.02		*p* = 0.002	*p* = 0.04			
Non Sup CCOM × OME	*p* = 0.02							
Non Sup CCOM × Non Sup COM							*p* = 0.04	
AOM × Non Sup COM							*p* = 0.02	

In addition, it was possible to observe worse bone thresholds in patients with tinnitus, in all frequencies analyzed (*p* < 0.05). We also observed worse air thresholds in the presence of tinnitus, with increasing statistical significance in the highest frequencies (4000, 6000 and 8000 Hz with *p* < 0.001).

## Discussion

Hearing loss secondary to inflammatory processes of the middle ear is a topic widely addressed in the literature, with descriptions of sensorineural hearing loss dating from 1941.^12^ However, it was only from the 1970s that it began to prove that sensorioneural loss after otitis media may be the result of a cochlear insult.^13^, ^14^, ^15^, ^16^ Although there are several articles in the literature addressing sensorineural losses in otitis media, there is still no study comparing this type of hearing loss in its various clinical presentations.

In our sample of 151 ears, we observed that conductive hearing loss corresponded to 58.28% of our sample and was more frequent in the non-suppurative COM and OME groups. As they are ears with lower inflammatory activity, there is a lower chance of intracochlear injury compared to the ears of the other groups. Mixed hearing loss occurred in 40.4% of all ears, and were more frequent in the groups Suppurative COM, cholesteatomatous COM (suppurative and non-suppurative) and AOM — more active groups from the inflammatory and infectious point of view. These findings are parallel in the literature: several studies have demonstrated, in temporal bones, that the degree of injury in the sensory structures of the cochlea has an intimate relationship to the degree and severity of inflammatory pathological changes in the middle ear.^10^, ^13^, ^14^, ^17^

In addition, two ears with AOM had pure sensorineural hearing loss at higher frequencies. This result goes according to Song et al.^18^ and Margolis and Nelson,^19^ who describe cases of pure sensorineural hearing loss after AOM. It is possible that the pathophysiological mechanism for these cases is the passage of harmful agents from the middle ear to the basal gyration of the cochlea through the membrane of the round window, considering the tonotopic relationship of this region with the frequencies reached. As reported in the report by Margolis and Nelson,^19^ at the beginning of the picture there was no effusion and, therefore, there was no presence of air-bone GAP.

Regarding symptomatology, tinnitus was the second largest complaint reported by patients. Tinnitus is more prevalent in the ears with a higher degree of infectious/inflammatory activity. This indicates a probable lesion to the cochlear neuroepithelium, as discussed by Tailor et al.,^20^ since tinnitus is more intense in groups with more severe inflammatory conditions. In addition, we found worse bone thresholds at 500–4000 Hz in patients with tinnitus when compared to patients without tinnitus. We also observed significantly worse air thresholds at frequencies of 4000 Hz, 6000 Hz and 8000 Hz in tinnitus patients. This leads us to believe that the worst air thresholds in acute frequencies in patients with tinnitus may reflect more intense neural injury in the basal spin of the cochlea.^21^

In relation to vocal audiometry, we found worse SRTs values in the groups with higher inflammatory/infectious activity. Only 10 ears of 151 (6.62%) had discrimination scores < 88%, and we could observe worse discrimination scores in the suppurative and cholesteatomatous COM (non-suppurative form). The fact that SRTs has greatly altered values between groups of otitis media compared to the low frequency of changes in the discrimination scores suggests that the cochlear lesion secondary to otitis media occurs primarily in sensory epithelium (hair cells) and, only in more severe and late cases, affect neural structures.^22^ Speech recognition changes are secondary to the degeneration of internal hair cells and neural cells, which occurs later.^23^ This situation is explained by the degeneration of external hair cells and vascular stria in comparison to the lesion in internal hair cells in the various forms of otitis media.^17^ The authors also show that the degree of injury to internal hair cells was closely correlated with the severity and chronicity of the inflammatory process in the middle ear, similarly to our clinical findings.

Moreover, it was possible to observe that the presence of tinnitus associated with significantly higher SRT values as compared with patients without tinnitus, as shown in Fig. 5. This finding indicates the presence of a cochlear lesion.^24^ Another possible mechanism for the onset of tinnitus would be that proposed by Liberman et al.,^25^ who demonstrated that otitis media and chronic hearing deprivation may occur with cochlear sinaptopathy and impairment of aferens and aferental innervation of hair cells.

Regarding tonal audiometry, the groups of otitis media showed worse air and bone thresholds in all frequencies, when compared with the ears of the control group (Fig. 7). In addition, it is possible to observe a descending characteristic of the thresholds, a fact also observed by other authors.^18^, ^26^, ^27^, ^28^ As the acute frequencies are the most affected, we can infer that the basal turn of the cochlea is the most affected by the infectious-inflammatory processes that occur in the middle ear.^10^, ^14^

We observed worse bone thresholds in the most active ears from the infectious and inflammatory point of view, such as ears with suppurative COM, cholesteatomatous COM and AOM. This finding corroborates that of English et al.,^9^ who found worse bone thresholds in patients with COM with discontinuity of the ossicular chain and with mastoid extension (more active COMs), when compared with the non-suppurative form of the COM. We did not observe a significant difference in air and bone thresholds in patients with suppurative COM and cholesteatomatous COM. That is, in our study cholesteatoma was not related to worse auditory results, as reported by other authors.^26^, ^27^, ^29^ In addition, there was no difference between the types of cholesteatoma (suppurative and non-suppurative) with regard to auditory thresholds. These findings suggest that auditory sequelae seem to be related to the local inflammatory process itself and bone destruction.

A curious observation of our study is that the ears with AOM had worse bone thresholds at 3000 and 4000 Hz. This is explained by the inflammatory lesion of the basal turn of the cochlea secondary to acute middle ear infection. It is proven that, in the acute situations, the membrane of the round window presents an increase in its permeability, with a subsequent decrease of its semipermeable nature in the chronicity of the picture.^7^, ^30^, ^31^ In addition, previous studies have shown that in the initial phase of AOM temporary functional changes may occur in the cochlea, called “Temporary Threshold Shifts” (TTS), which may recover after resolution of the acute infectious process.^32^, ^33^ According to our methodology, we included patients with AOM of up to 5 days of evolution, which may have led to these observed results.

In addition, it was possible to observe worse bone thresholds with advancing age in chronic cases of otitis media, in agreement with several authors.^29^, ^34^ Probably chronic hearing deprivation,^25^ acute episodes of AOM and exacerbations of COM over the years lead to cochlear insults, which can add to age-related auditory degeneration. The latter factor influences bone audiometric thresholds, because we observed that older individuals with normal ears have higher bone-based auditory thresholds. However, it is noted that bone auditory thresholds are within normal limits, as they do not exceed 25 dBNA.

We did not observe a correlation between disease duration and worse bone thresholds in the ears with COM and OME (*p* = 0.1362), in agreement with some authors.^11^, ^27^ However, the relationship of duration of the disease and increase of bone thresholds has been previously reported.^28^, ^34^, ^35^ Perhaps our results did not point to such correlation due to a small sample of patients in each group. Another hypothesis for the lack of correlation would be the probable thickening of the round window membrane, acting as a protective factor for the cochlea against inflammatory aggressions of the middle ear.^7^

Our study has some limitations. Although we have a group of 116 patients, the number of ears in each of the otitis media subgroups was limited. Moreover, because it is a cross-sectional study, we could not follow the auditory evolution with the treatment (clinical or surgical), as well as we could not define whether the hearing loss is temporary or permanent.

Despite the limitations, this is the first study to compare the auditory thresholds in the different clinical presentations of otitis media. We adopted many exclusion criteria, being very careful in the selection of our patients. We chose to include children over 10 years of age, who are more collaborative, contributing to more reliable responses during the audiometry exam. Moreover, at this age, the auditory system already has greater development.^36^ We excluded patients who underwent previous otological surgery, due to the increased risk of sensorineural hearing loss after such surgeries.^36^, ^37^ As our exclusion criteria were very strict, there was a reduction in patients who could participate in the study. In addition, all our exams were performed by an experienced audiologist, ensuring a reliable result.

## Conclusion

Our results confirm the auditory impairment (airway and bone) in all types of otitis when comparing them with the control ears. Bone hearing loss is more pronounced in patients with a higher degree of suppuration and in acute conditions, especially at higher frequencies.

## Conflicts of interests and source of funding

We wish to confirm that there are no known conflicts of interest associated with this publication and there has been no significant financial support for this work that could have influenced its outcome. We confirm that the manuscript has been read and approved by all named authors and that there are no other persons who satisfied the criteria for authorship but are not listed.

Ana Luiza Papi Kasemodel de Araujo, Francisco Polanski Cordeiro, and Rafael da Costa Monsanto received a scholarship from the Coordenação de Aperfeiçoamento Pessoal de Nível Superior (CAPES) (Finance code: 001). Rafael da Costa Monsanto is part of a research project funded by NIH NIDCD U24 1U24DC020851-01, International Hearing Foundation, and Lions 5M International. Norma de Oliveira Penido is funded by Fundação de Amparo a Pesquisa do Estado de São Paulo – FAPESP process number 2021/07346-6 and Council for Scientific and Technological Development – CNPq process number 302910/2020-4.
